# 3‐dimensional visualization of implant‐tissue interface with the polyethylene glycol associated solvent system tissue clearing method

**DOI:** 10.1111/cpr.12578

**Published:** 2019-02-03

**Authors:** Yating Yi, Yi Men, Dian Jing, Wenjing Luo, Shiwen Zhang, Jian Q. Feng, Jin Liu, Woo‐Ping Ge, Jun Wang, Hu Zhao

**Affiliations:** ^1^ State Key Laboratory of Oral Diseases, West China School of Stomatology Sichuan University Chengdu China; ^2^ Department of Restorative Sciences, College of Dentistry Texas A&M University Dallas Texas; ^3^ Department of Biomedical Sciences, College of Dentistry Texas A&M University Dallas Texas; ^4^ Laboratory of Stem Cell Biology, State Key Laboratory of Biotherapy, West China Hospital Sichuan University Chengdu China; ^5^ Children’s Research Institute University of Texas Southwestern Medical Centre Dallas Texas

**Keywords:** angiogenesis, implant, osseointegration, PEGASOS, tissue clearing

## Abstract

**Objectives:**

Dental implants are major treatment options for restoring teeth loss. Biological processes at the implant‐tissue interface are critical for implant osseointegration. Superior mechanical properties of the implant constitute a major challenge for traditional histological techniques. It is imperative to develop new technique to investigate the implant‐tissue interface.

**Materials and methods:**

Our laboratory developed the polyethylene glycol (PEG)‐associated solvent system (PEGASOS) tissue clearing method. By immersing samples into various chemical substances, bones and teeth could be turned to transparent with intact internal structures and endogenous fluorescence being preserved. We combined the PEGASOS tissue clearing method with transgenic mouse line and other labelling technique to investigate the angiogenesis and osteogenesis processes occurring at the implant‐bone interface.

**Results:**

Clearing treatment turned tissue highly transparent and implant could be directly visualized without sectioning. Implant, soft/hard tissues and fluorescent labels were simultaneously imaged in decalcified or non‐decalcified mouse mandible samples without disturbing their interfaces. Multi‐channel 3‐dimensional image stacks at high resolution were acquired and quantified. The processes of angiogenesis and osteogenesis surrounding titanium or stainless steel implants were investigated.

**Conclusions:**

Both titanium and stainless steel implants support angiogenesis at comparable levels. Successful osseointegration and calcium precipitation occurred only surrounding titanium, but not stainless steel implants. PEGASOS tissue clearing method provides a novel approach for investigating the interface between implants and hard tissue.

## 1 INTRODUCTION

Dental and orthopaedic implants have revolutionized the treatment of patients with missing teeth or damaged bones and joints.^1^ Success of implant placement is determined by their interactions with the host tissue occurred mostly at the implant‐tissue interface.^2^, ^3^ Among the many biological processes of implant‐tissue interface being evaluated, angiogenesis and osteogenesis are the two most important ones.^4^, ^5^, ^6^ Implants interact with local vasculature and regulate the production of endothelial progenitor cells to form new blood vessels, which bring in oxygen, nutrition and stem cells to the interface.^7^ Although a large number of studies have confirmed the close association between angiogenesis and osteogenesis during osseointegration process,^8^, ^9^ direct visualization of the two processes simultaneously remained extremely challenging due to limited research approaches.

Growing evidence has suggested that interplays between blood vessels with bone tissue occur in 3‐dimensions and are essential for regulating local stem cell populations and various signal pathways.^10^, ^11^ Multiple transgenic mouse models have been developed to label different types of tissues with endogenous fluorescence, including GFP and tdTomato.^12^ These mouse models provide possibility to investigate multiple events occurred at the implant‐bone interface concomitantly.

Histological sectioning methods remain as the golden standards for visualization and quantitative measurement of peri‐implant angiogenesis and new bone formation.^9^, ^13^ During the sample preparation, implants were removed to enable tissue embedding and sectioning,^14^ which inevitably destroyed the integrity of the interface.^15^ Alternatively, ground sections were employed to study implant‐bone interface without decalcification treatment or removing implants.^15^, ^16^ Although calcein green dynamic labelling and several other histology staining could be performed, the hard tissue embedding process quenched endogenous fluorescence and compromised most immunofluorescence staining signals.^17^, ^18^ In addition, only very few ground sections could be achieved from a sample, which provided very limited information for the whole tissue.^19^, ^20^ µCT remains to be the only available approach to investigate an intact bone‐implant interface in 3‐dimension. It cannot visualize cellular component or fluorescent signals.

Another technical challenge for studying implant‐tissue interface is quantification. Implant‐tissue interface occurs in complicated three‐dimensional form. Sectioning image provides only 2‐D information. Quantification results vary significantly depending on sectioning and sampling locations. In contrast, 3‐D imaging provides spatial information of the whole sample and quantification result is more faithful and comprehensive.

Tissue clearing technique enables deep 3‐D imaging of tissues with a confocal, two‐photon or light‐sheet microscope by turning them transparent.^21^, ^22^, ^23^, ^24^, ^25^, ^26^ Hard tissues are opaque because of mismatched refractive index (RI) among various components, including minerals, lipids, pigments and water. All tissue clearing methods followed similar principle which is to remove components blocking or diffracting the light. Transparency can finally be achieved after the tissue interstitial fluid is replaced with clearing medium with consistent RI.^27^ Once the transparency being reached, images can be acquired even at several millimetres depth with high resolution.

Current tissue clearing methods can be classified into three major categories: (a) Organic solvent‐based clearing methods, including DISCO series,^23^, ^28^, ^29^ Fluoclear^22^ and polyethylene glycol (PEG)‐associated solvent system (PEGASOS).^30^ (b) Aqueous reagent‐based clearing methods, including Scale,^31^ ClearT,^32^ SeeDB,^33^ CUBIC series.^25^, ^34^, ^35^ (c) Hydrogel‐based clearing methods, including CLARITY,^24^ PACT.^21^ Three major criteria to evaluate a clearing method include transparency outcome, fluorescent preservation and applicability of tissues. Overall, solvent‐based methods achieve better transparency than other types. Aqueous methods achieve better fluorescence preservation than other methods. Several methods have been developed for clearing hard tissues, including PACT‐deCAL,^21^ mPACT,^36^ CUBIC,^25^ Bone CLARITY,^37^ PEGASOS^30^ and vDISCO.^28^ Among them, PEGASOS has its unique advantages as it achieved favourable transparency of both soft and hard tissues with fluorescence preservation, relatively short time and low cost. Intact mouse head, mandible bone with teeth, knee joint and long bone could be imaged with a two‐photon microscope after tissue clearing process without sectioning.^30^

In the current study, we introduced the application of the PEGASOS method on studying the implant‐bone interface. A transgenic mouse model, *Cdh5‐Cre^ERT2^*; *Ai14* mouse line, was used to label blood vessels specifically. Angiogenesis and osteogenesis at the interface of stainless steel (SS) implants or titanium (Ti) implants were investigated. We demonstrated that 3‐D imaging based on PEGASOS tissue clearing method is a useful new tool for investigating the implant‐tissue interface.

## 2 MATERIALS AND METHODS

### 2.1 Animal breeding and tamoxifen induction

All protocols for animal care and experiments were reviewed and approved by the Institutional Animal Care and Use Committee at Texas A&M University, College of Dentistry. *Cdh5‐Cre^ERT2^*mice^38^ (provided by Dr Woo‐Ping Ge in the UTSW with MTA form approved by Cancer Research Limited) were crossed with *Ai14 *(JAX 007908) reporter mice. Tamoxifen (Sigma Aldrich, St. Louis, MO, USA, Sigma Prod. No. T5648) dissolved in corn oil was injected intraperitoneally for adult *Cdh5‐Cre^ERT2^*; *Ai14* mice (6‐8 weeks of age) with dosage of 9 mg/40 g body weight daily for 2 days.

### 2.2 Implant placement surgery

*Cdh5‐Cre^ERT2^*; *Ai14* mice at 6‐8 weeks of age were anaesthetized with an intraperitoneal injection of ketamine (100 mg/kg) and xylazine (10 mg/kg). Mandibular first molars were extracted with forceps. Following that, titanium implant (0.6‐mm‐diameter titanium dentine pins, STABILOK) or stainless steel dentin pin (0.6‐mm‐diameter stainless steel dentin pins, STABILOK) was screwed into the extraction socket with ~1.5 mm depth and was cut at the level of gingiva level.

### 2.3 μCT analysis

Mandible samples were placed in a 12.3 mm tube, and μCT scanning was performed using a SCANCO μCT35 device at Texas A&M University, College of Dentistry. The μCT images were acquired with the X‐ray source at 70 kV voltage. The data were collected at a voxel size of 7 μm resolution. The reconstruction of 3‐D images was performed with imaris 9.0 (Bitplane, AG, Zurich, Switzerland).

### 2.4 PEGASOS tissue clearing process

Polyethylene glycol‐associated solvent system tissue clearing was performed as previously described.^30^ Mice were transcardially perfused with 50 mL heparin‐PBS (10 U/ml heparin sodium in 0.01 M PBS) and 20 mL 4% PFA (4% paraformaldehyde in 0.01 M PBS, pH 7.4). Samples were then fixed in 4% PFA overnight at room temperature. For decalcification treatment, mandibles were then immersed in 20% *w/v* EDTA (pH 8.0) (Sigma‐Aldrich; E9884) solution at 37°C for 4 days and EDTA solution was refreshed daily. For non‐decalcified PEGASOS, above EDTA treatment was skipped. Next, samples were decolourized with 25% (*v*/*v* in H_2_O) Quadrol (Sigma‐Aldrich; 122262) solution for 2 days at 37°C to remove blood heme under constant shaking. Serial delipidation was then performed at 37°C under constant shaking for 6 hours/solution: 30% tert‐Butanol (tB, Sigma‐Aldrich; 471712) solution, 50% tB solution and 70% tB solutions. 30% tB solution is composed of 70% *v*/*v* H_2_O, 27% *v*/*v* tB and 3% *w/v* Quadrol. 50% tB solution is composed of 50% *v*/*v* H_2_O, 47% *v*/*v* tB and 3% *w*/*v* Quadrol. 70% tB solution is composed of 30% *v*/*v* H_2_O, 67% *v*/*v* tB and 3% *w*/*v* Quadrol. Following delipidation, samples were dehydrated in tB‐PEG solution composed of 70% tB, 27% (*v*/*v*) poly(ethylene glycol) methyl ether methacrylate average Mn500 (PEG MMA500) (Sigma‐Aldrich; 447943) and 3% (*w*/*v*) Quadrol at 37°C. Finally, samples were immersed in the BB‐PEG clearing medium which is composed of 75% (*v*/*v*) benzyl benzoate (BB) (Sigma‐Aldrich; B6630), 22% (*v*/*v*) PEG MMA500 and 3% (*w*/*v*) Quadrol. Complete transparency could be achieved usually within 24 hours. Samples could be preserved in the BB‐PEG clearing medium at room temperature for storage and imaging.

### 2.5 Imaging acquisition

Fluorescent images of mouse mandible or peri‐implant region were acquired with (ZEISS, Oberkochen, Germany) LSM 780 Upright (ZEISS) or ZEISS LSM 880 Inverted two‐photon microscopy. Following objectives were used for our study. A 10×/0.3NA objective (ZEISS; EC Plan‐Neofluar, 10×/0.3, working distance: 5.2 mm) was used on either microscope; a 25×/0.8NA multi‐immersion objective (ZEISS; LD LCI Plan‐Apochromat 25×/0.8 lmm Corr DIC M27 for oil, water or glycerine immersion, working distance: 0.57 mm) was used on ZEISS LSM 780 Upright. Fluorescence images of 4 channels were captured sequentially with BP420‐480, BP500‐550 filters. A 561 nm excitation laser line and emission between 570 and 630 nm were used for acquiring tdTomato signal. A 488 nm excitation laser line and emission between 504 and 556 nm were used for acquiring calcein green signal. Reflection light was triggered by 16% of 488 nm laser. Coherent Chameleon Ultra II Ti:sapphire laser at 950 nm wavelength and non‐descanned detector were used for second harmonic signals (SHG) imaging.

For 10×/0.3 objective, image stacks were acquired at 1024 × 1024 pixels resolution (pixel size 1.19 μm) with 5 μm *z*‐step. With 25×/0.8NA objective, images stacks were acquired at 1024 × 1024 pixels resolution (pixel size: 0.474 μm) with a 2 μm *z*‐step. Deconvolution was performed with Autoquant X3 (Media Cybernetics, Rockville, MD, USA) by using blind deconvolution parameter set‐up.

### 2.6 3‐D reconstruction of images

Individual channels were merged with imagej (NIH, Bethesda, MD, USA). Three‐dimensional reconstruction and quantitative analysis were performed with imaris 9.0 (Bitplane). When performing reflection imaging, non‐specific reflecting signal was also detected in bone tissue. To remove the background noise, reflection signal from the implant on each optical slice was manually outlined to create a Surface. The reflection light channel was masked under the created “Surface,” and a new individual channel was created to depict only the implant without background noise. Image stacks were reconstructed using the “volume rendering” function. Snapshot was generated using the “Snapshot” function. Movies were generated using the “Animation” function.

### 2.7 Quantitative analysis of blood vessels volumes and bone volume

Quantitative analysis was performed with imaris 9.0 (Bitplane). A 150 × 150 × 150 μm stack in the thread grooves of each implant was defined as region of interest (ROI). Only the channel representing blood vessels was involved in analysis. Volume of selected blood vessels in the ROI was quantified using “Statistics” function. For each sample, at least four randomly selected ROIs were selected for quantification.

Bone volume was quantified based on the Second Harmonic Generation signal (SHG). ROI was generated in the SHG signal channel. Volume of selected structure in ROI was quantified using “Statistics” function.

### 2.8 Analysis of vasculature‐implant direct contact points

Stacks of 150 μm thickness containing both tdTomato and reflection signal channels are selected near the implant groove surface. Direct contact between blood vessels and implant can be visualized on individual optical slice. The number of direct contact points can then be measured for the entire image stack.

### 2.9 BV/TV quantification

Bone volume/total volume (BV/TV) quantification in μCT image data was performed with imaris 9.0. BV/TV was defined as: the volume of selected high‐density region/total volume of ROI.

### 2.10 Statistic analysis

N numbers are displayed in the figures. Data are presented as mean ± standard deviation using Student’s *t* tests or one‐way ANOVA. Statistical analysis was performed with Microsoft Excel and GraphPad Prism.

### 2.11 Data availability

The data that support the findings of this study are available from the corresponding author, HZ, upon reasonable request.

## 3 RESULTS

### 3.1 PEGASOS tissue clearing method efficiently renders mandible bones and teeth transparent

*Cdh5‐Cre^ERT2^*; *Ai14* mouse model was used for implant placement (Figure S1a). Tamoxifen induction was performed for adult mice of 6‐8 weeks of age. Based on previous experience, all blood vessels including arteries, veins and capillaries can be labelled within 1 week after tamoxifen induction.^38^ Implant placement surgery was performed 1 week after induction（Figure S1b‐d）. Soft tissue healed rapidly 1 week after surgery and formed peri‐implant sealing（Figure S1e）. Mice with mandibles were sacrificed at 1, 7, 14 or 21 days after surgery (Figure S1a).

Mice mandibles were collected and processed following PEGASOS method with or without decalcification treatment (Figure S2a,b). Clearing with decalcification achieved better transparency. Mandibles and teeth were nearly invisible, and implant could be directly visualized (Figure S2c,d). Partial transparency was achieved without decalcification. Mandible was partially transparent, but teeth were not. Implant could still be clearly visualized. (Figure S2e,f). To investigate whether clearing treatment compromises bone structures, µCT images were acquired before and after clearing treatment (without decalcification; Figure S2g,h). Overlaid image showed no detectable difference, indicating clearing treatment had no significant impact on bone organization (Figure S2i).

### 3.2 PEGASOS‐based deep imaging acquires multi‐channel images of implant‐tissue interface with high resolution

**Figure 1:**
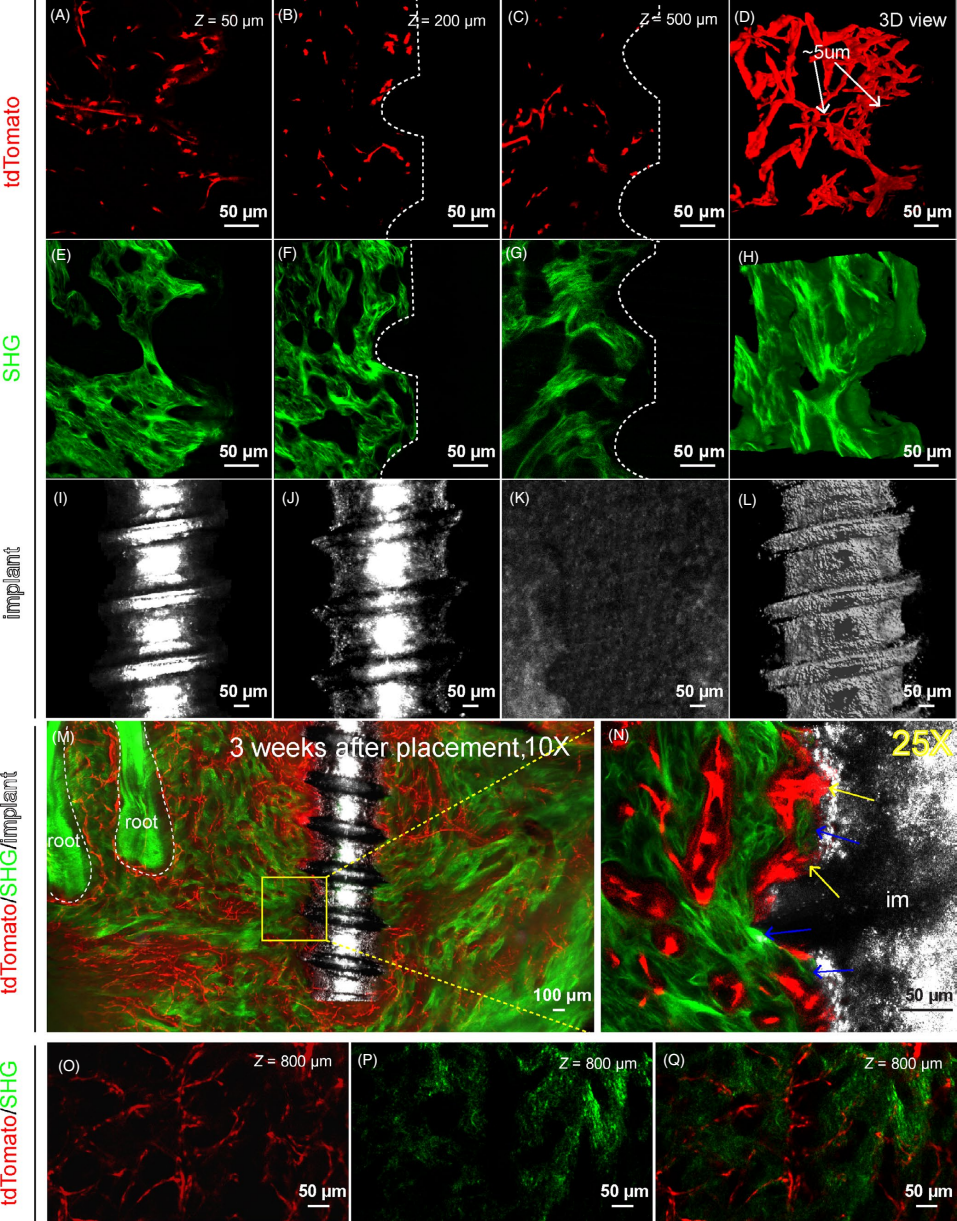
Polyethylene glycol (PEG)‐associated solvent system (PEGASOS)‐based deep imaging enables multi‐channel imaging of implant‐tissue interface with high resolution. Adult *Cdh5‐Cre^ERT2^*; *Ai4* mice (6 wk of age) were used for titanium implant placement. Mandible samples were processed following PEGASOS method with decalcification treatment. Images were acquired with 25× (working distance 0.57 mm) (panels A‐L, N) or 10× (working distance 5.2 mm) (panels M, O, P, Q) objective. A‐C, Optical sections of tdTomato signal displaying blood vessels (tdTomato) near the implant at the depth of 50 μm (A), 200 μm (B) or 500 μm (C). D, 3‐D view of a 150‐μm‐thick optical stack of tdTomato signal displaying blood vessels near the implant. Arrows show capillaries with the diameter of ~5 μm. E‐G, Optical sections of second harmonic generation (SHG) signal displaying bone near implants at the depth of 50 μm (E), 200 μm (F) or 500 μm (G). H, 3‐D view of a 150‐μm‐thick optical stack of SHG signal displaying bone tissue near the implant. I‐K, Optical sections of reflection signal displaying implants at the depth of 50 μm (I), 200 μm (J) or 500 μm (K). No signal was detected at depth of 500 μm (K) because the reflection signal could not pass through the diameter of the implant. L, 3‐D view of a 150‐μm‐thick optical stack of reflection signal showing the implant. M, A 400‐μm‐thick optical stack showing a titanium implant within the mandible bone with surrounding vasculature. N, Optical section of boxed area in (M) was acquired with a 25× objective. Yellow arrows show blood vessels in direct contact with the implant surface. Blue arrows indicate direct bone‐implant contact. O‐Q, Optical sections showing blood vessels (O) and bone (P) at the depth of 800 μm in the mandibular furcation region. Scale bars, 100 μm in panel M, 50 μm in other panels. im, implant

Next, we tested appropriate conditions for imaging endogenous fluorescence, non‐fluorescent bone tissue and implant. Confocal or two‐photon microscope was used for our study. Adult *Cdh5‐Cre^ERT2^*; *Ai14* mice were induced with tamoxifen, and titanium implant was placed. Samples were collected 1 month later for processing and imaging. Three‐channel image stacks of 500 µm thickness near the implant were acquired with 25×/0.8NA objective (Figure 1A‐C,E‐G,I‐K). TdTomato signal labelled all the blood vessels. After clearing with PEGASOS method with decalcification treatment, signals of tdTomato could be clearly detected at 50, 200 or 500 μm depth (Figure 1A,B,C). A 3‐D reconstruction displayed spatial organization of blood vessels including capillaries with diameter of no more than 5 μm (Figure 1D). SHG signal arises from type I collagen which is enriched in mature bone and dental tissues. SHG signal could be clearly detected at 50, 200 or 500 μm depth (Figure 1D,F,G). 3‐D reconstruction displayed spatial organization of alveolar trabecular bone (Figure 1H). Reflection signal was used to image the implant surface (Figure 1I‐L). We used 488 nm laser as the reflection excitation light. The outline of implant could be clearly identified in optical slices at 50 or 200 μm depth (Figure 1I,J). The diameter of the implant is 600 μm, and reflected light could not pass through the radius position. Therefore, no signal was detected beyond 500 μm depth (Figure 1K). 3‐D reconstruction of the reflection signal depicted half side of the implant surface (Figure 1L, Movie S1). Working distance of ZEISS 25×/0.8NA objective is no more than 500 μm. When using a 10×/0.3NA objective with 5.2 mm working distance, tdTomato and SHG signal could be detected as deep as 800 μm (Figure 1O‐Q).

We imaged a 3413 × 2317 × 400 μm volume with a 10×/0.3NA objective using a two‐photon microscope. Alveolar bone and dental root, which were enriched with Collagen I, could be detected with SHG. Enriched blood vessels were detected surrounding the implant and within the bone marrow space (Figure 1M). Selected area near the implant surface was re‐imaged with a 25×/0.8NA objective. Enriched blood vessel and bone were clearly detected on the implant surface with direct contacts with the implant surface (arrows in Figure 1N, Movie S2).

### 3.3 PEGASOS clearing process preserves intact bone‐implant interface

**Figure 2:**
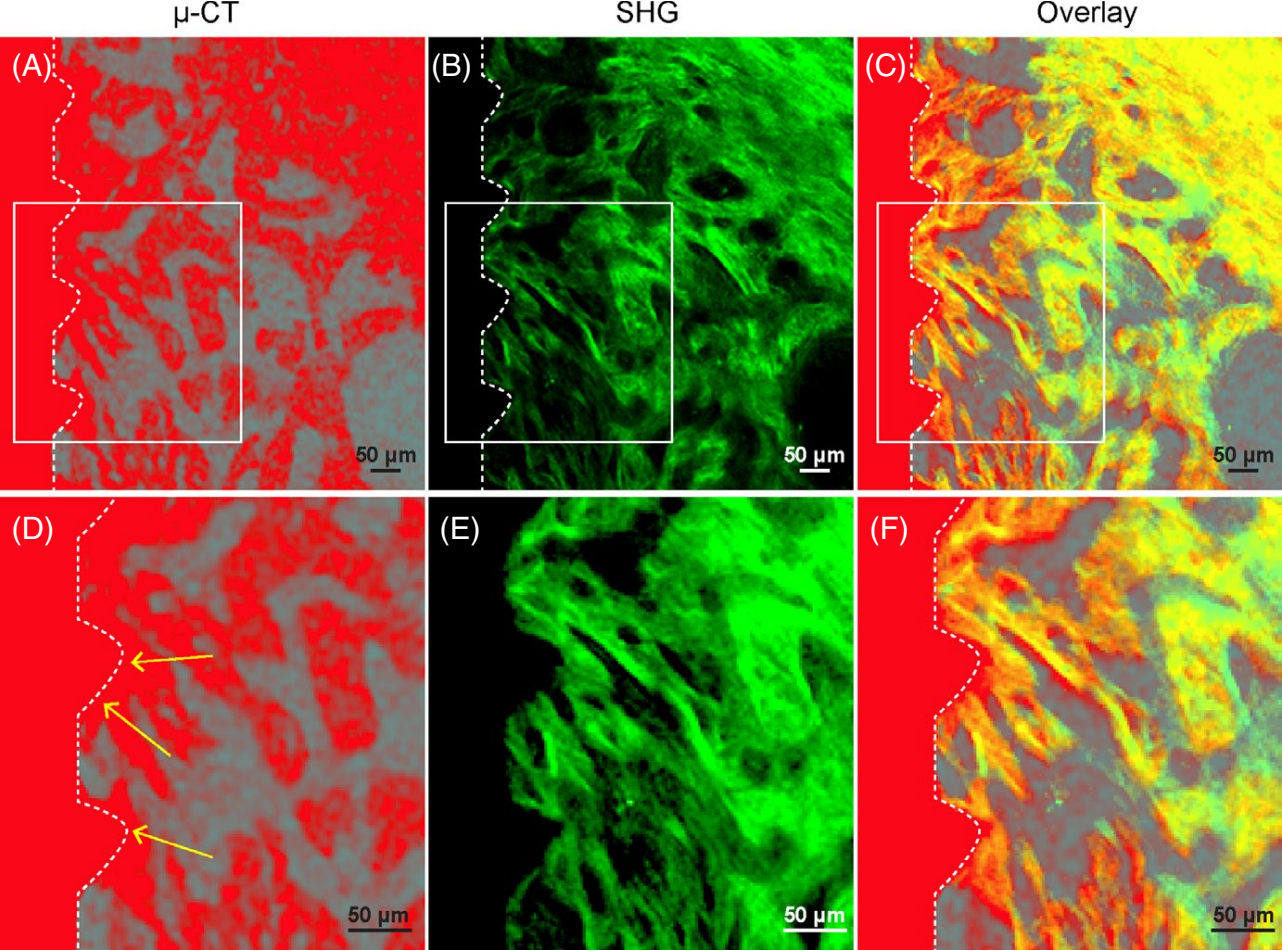
Second harmonic generation (SHG) signal presents intact bone microstructure at the implant‐bone interface without artefact. Wild‐type mice were used for implant placement. Mandible samples were collected 1 mo after placement and processed following polyethylene glycol (PEG)‐associated solvent system clearing protocol with decalcification treatment. A, μCT image of the bone‐implant interface acquired before decalcification. High‐density bone and implant were shown in red colour. B, SHG signal (green) was acquired after clearing with a two‐photon microscope at identical position as in panel A. C, Overlay of (A) and (B). D‐F, Boxed area in (A), (B) and (C) were enlarged. Arrows in d indicate halation artefact of μCT. Scale bars, 50 μm

It remains controversial whether decalcification compromises the bone‐implant interface. In addition, it remains unknown whether SHG signal can truly display the bone structure with sufficient details. To test this, we harvested a mandible sample 1 month after titanium implant placement. μCT images were acquired prior to clearing process. Next, the sample was cleared following the PEGASOS decalcification method and SHG signal was imaged. We were able to locate an identical anatomical region in both μCT and SHG image dataset for comparison. Both μCT and SHG signal revealed trabecular bone organization near the implant surface (Figure 2A,B). Overlaying the two images showed complete overlapped details from the two datasets (Figure 2C). Boxed area on the implant surface was zoomed in. In μCT image, bone on the implant surface could not be distinguished from the implant due to metal halation artefact (Figure 2D).^39^, ^40^ In contrast, SHG signal image showed no such artefact and the boundary between bone and implant could be clearly identified (Figure 2E). Overlaying of the two images indicates nearly all structures revealed with μCT analysis were also displayed in SHG image (Figure 2F). In summary, PEGASOS decalcification method preserves intact bone‐implant interface and SHG signal reveals better bone tissue details than μCT analysis.

### 3.4 PEGASOS renders non‐decalcified mandibles partially transparent and enables visualization of calcein green signal

**Figure 3:**
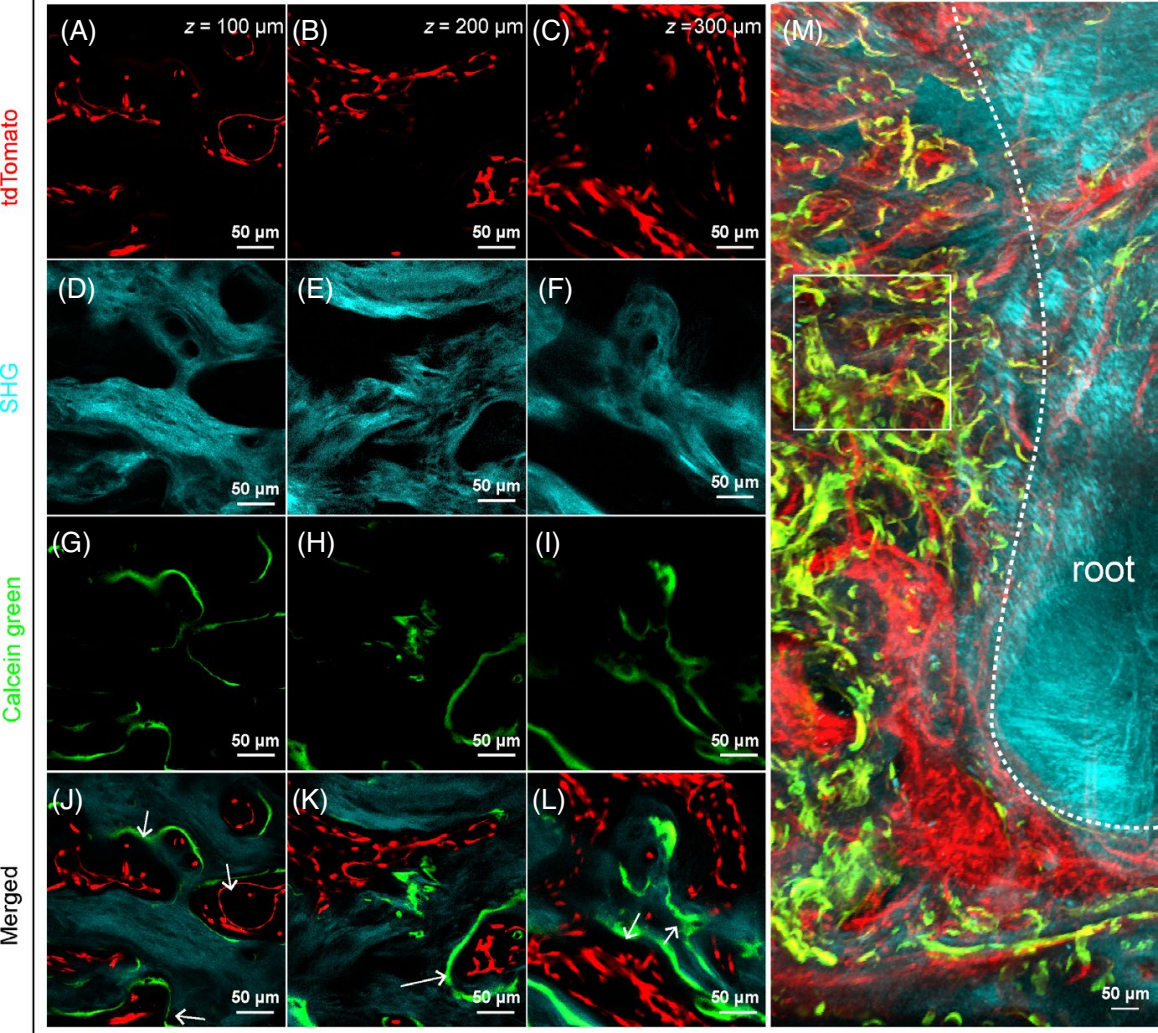
Polyethylene glycol (PEG)‐associated solvent system (PEGASOS) clearing without decalcification treatment enables visualization of calcein green labelling signal. Adult *Cdh5‐Cre^ERT2^*; *Ai4* mice (2 mo of age) were induced with tamoxifen and calcein green was injected. Mandible samples were processed following PEGASOS method without decalcification treatment. Images were acquired with a 25× objective on a ZEISS two‐photon microscope. A‐C, Optical sections of tdTomato signal showing blood vessels in the furcation region at the depth of 100 μm (A), 200 μm (B) or 300 μm (C). D‐F, Optical sections of second harmonic generation signal showing bone at the depth of 100 μm (D), 200 μm (E) or 300 μm (F). G‐I, Optical sections of calcein green signal showing calcium precipitation at the depth of 100 μm (G), 200 μm (H) or 300 μm (I). J‐L, Merged images of three channels. White arrows indicate calcium precipitation occurs exclusively surrounding blood vessels. M, 3‐D optical stack of ~150 μm thickness showing active calcium precipitation (green) and blood vessels (red) near the root surface (cyan). Box indicates approximate position where panels A‐L were acquired. Scale bars, 50 μm

Calcein green labelling is a routine technique for investigating dynamic osteogenic activity of the bone tissue by detecting their calcium precipitation and is not compatible with decalcification treatment.^41^ Mandible bones cleared with PEGASOS method without decalcification achieved only partial transparency. We tested whether calcein green labelling signal can be visualized in 3‐D. Adult *Cdh5‐Cre^ERT2^*; *Ai14* mice of 6‐8 weeks of age were induced with tamoxifen. Seven days later, calcein green was injected and mice were sacrificed 24 hours later. Mandibles were processed following PEGASOS without decalcification procedure. Samples were imaged with a 10×/0.3NA objective on a two‐photon microscope. Optical slices of tdTomato signal (Figure 3A‐C), SHG signal (Figure 3D‐F) and calcein green signal (Figure 3G‐I) were acquired at 100, 200 and 300 μm depth. Signal quality deteriorated significantly when the depth is over 350 μm (data not shown). Images with merged three channels at 100, 200 and 300 μm depth clearly showed that calcium precipitation represented by calcein green signal is exclusively surrounding the vasculature within the bone marrow space (Figure 3J,K,L). A 3‐D image stack acquired near the root clearly showed enriched vasculature and active osteogenic activity adjacent to the root (Figure 3M). These results indicate that, despite reduced tissue transparency, PEGASOS clearing method without decalcification treatment still enables 3‐D imaging of calcein green labelling together with other labels.

### 3.5 Deep imaging of cleared mandible bone samples with decalcification revealed angiogenesis and osteogenesis processes at the titanium implant‐bone interface

**Figure 4:**
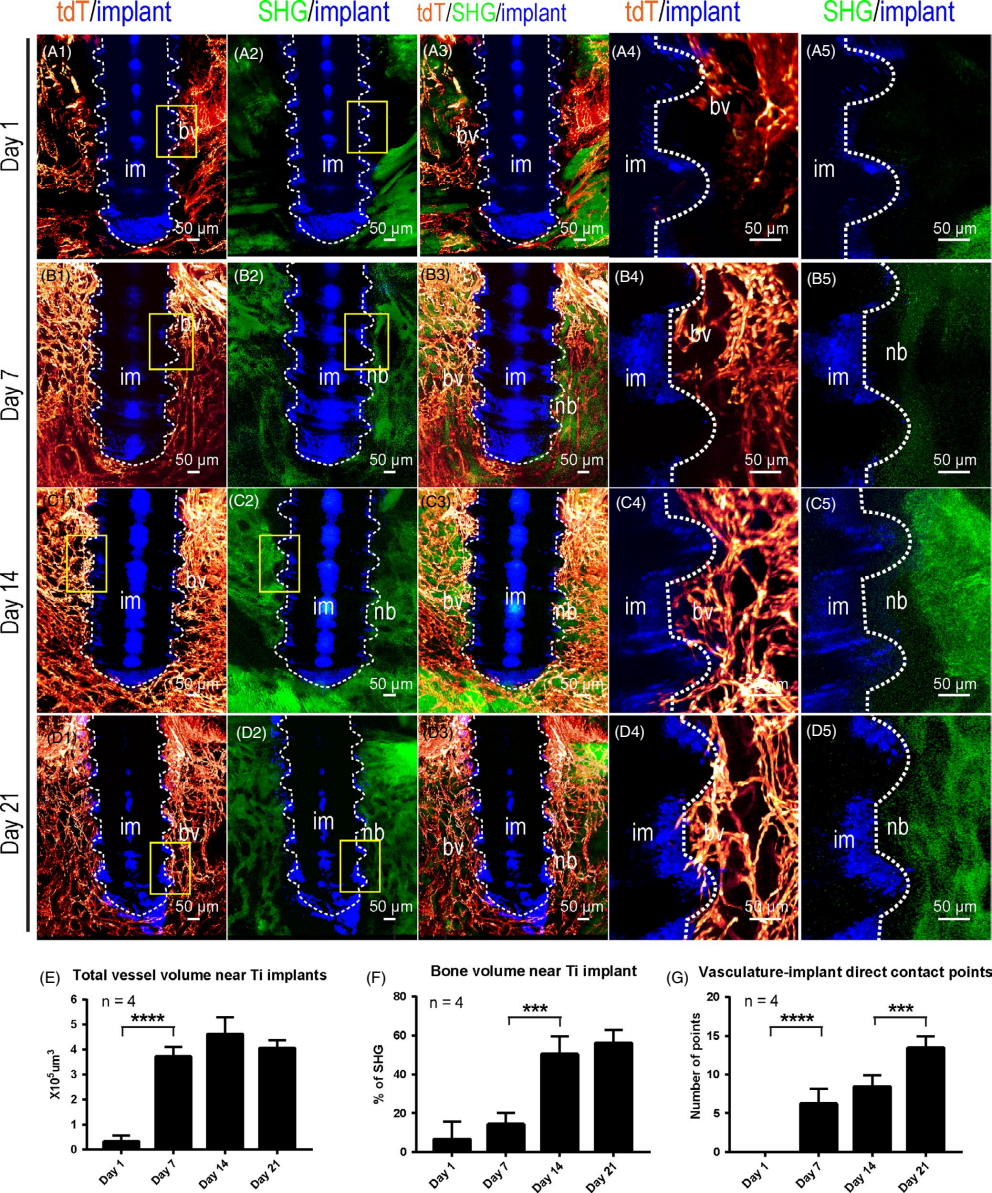
Three‐dimensional analysis indicates the progressive angiogenesis and osteogenesis on the surface of titanium implants during the osseointegration process. Adult *Cdh5‐Cre^ERT2^*; *Ai4* mice (2 mo of age) were used for titanium implant placement. Samples cleared with decalcified polyethylene glycol (PEG)‐associated solvent system method were imaged with a two‐photon microscope. Optical stacks of 150 μm thickness were acquired to demonstrate blood vessels (tdTomato, red) surrounding the interface between implant (reflection image, blue) and mandible bone (second harmonic generation, green). A1‐A3, Images were acquired with 10× objective 1 d after implant placement. A4‐A5, Boxed areas in A1 and A2 were re‐imaged with a 25× objective. A1‐A3, Images were acquired with 10× objective 1 wk after implant placement. B4‐B5, Boxed areas in B1 and B2 were re‐imaged with a 25× objective. C1‐C3, Images were acquired with 10× objective 2 wk after implant placement. C4‐C5, Boxed areas in C1 and C2 were re‐imaged with a 25× objective. D1‐D3, Images were acquired with 10× objective 3 wk after implant placement. D4‐D5, Boxed areas in D1 and D2 were re‐imaged with a 25× objective. E, Total blood vessels volumes in a 150 × 150 × 150 μm image stack near the implant surface at different time points were quantified, n = 4. F, Percentiles of bone tissue volume in a 150 × 150 × 150 μm image stack near the implant surface at different time points were quantified, n = 4. G, Numbers of direct contact points between the implant and blood vessels at various time points were quantified, n = 4. Scale bars, 100 μm. bv, blood vessels; im, implant; nb, new bone

Next, we investigated the osteogenesis and angiogenesis processes at the titanium implant‐bone interface. On the next day of the titanium implant placement surgery (Day 1), few blood vessels were detected near the implant surface (Figure 4A1,A3). Little SHG signal was detected near the implant surface (Figure 4A2,A3). Enlarged images confirmed the lack of vasculature and bone near the implant surface (Figure 4A4,A5). One week after procedure, significant amount of blood vessels was visualized within the implant thread grooves. Direct contacts were visualized between blood vessels and implant surface (Figure 4B1,B3,B4). Little bone was detected in the thread grooves of the implant at this time point (Figure 4B2,B3,B5). Two weeks after surgery, more blood vessels were detected within the thread grooves. Most of them were 2‐5 μm in diameter and contacted directly with the implant surface (Figure 4C1,C3,C4). Stronger SHG signal within the thread grooves was detected, suggesting more new bone formation within the thread grooves (Figure 4C2,C5). Three weeks after surgery, blood vessels were much enriched within the thread groove. Plenty of contacts were observed between blood vessels and the implant surface (Figure 4D1,D3,D4). Stronger SHG signal was detected in direct contact with the implant. Woven bone organization was observed, suggesting successful osseointegration of the implant (Figure 4D2,D5). The vasculature volume, bone volume and vasculature‐implant contact points were quantified with imaris 9.0 software based on 3‐dimensional images (Figure 4E‐G). Quantified analysis confirmed our previous observation. The bone volume changes at 1, 7, 14 or 21 days after titanium implant placement were also demonstrated with μCT analysis, which showed a similar trend after implant placement (Figure S3a‐d). All the results indicated that angiogenesis occurs prior to osteogenesis and takes no more than 2 weeks to complete after titanium implant placement. Osteogenesis occurs following angiogenesis and takes around 3 weeks to form implant osseointegration.

### 3.6 Angiogenesis surrounding stainless steel implants progressed normally, but osteogenesis progressed more poorly than surrounding titanium implants

**Figure 5:**
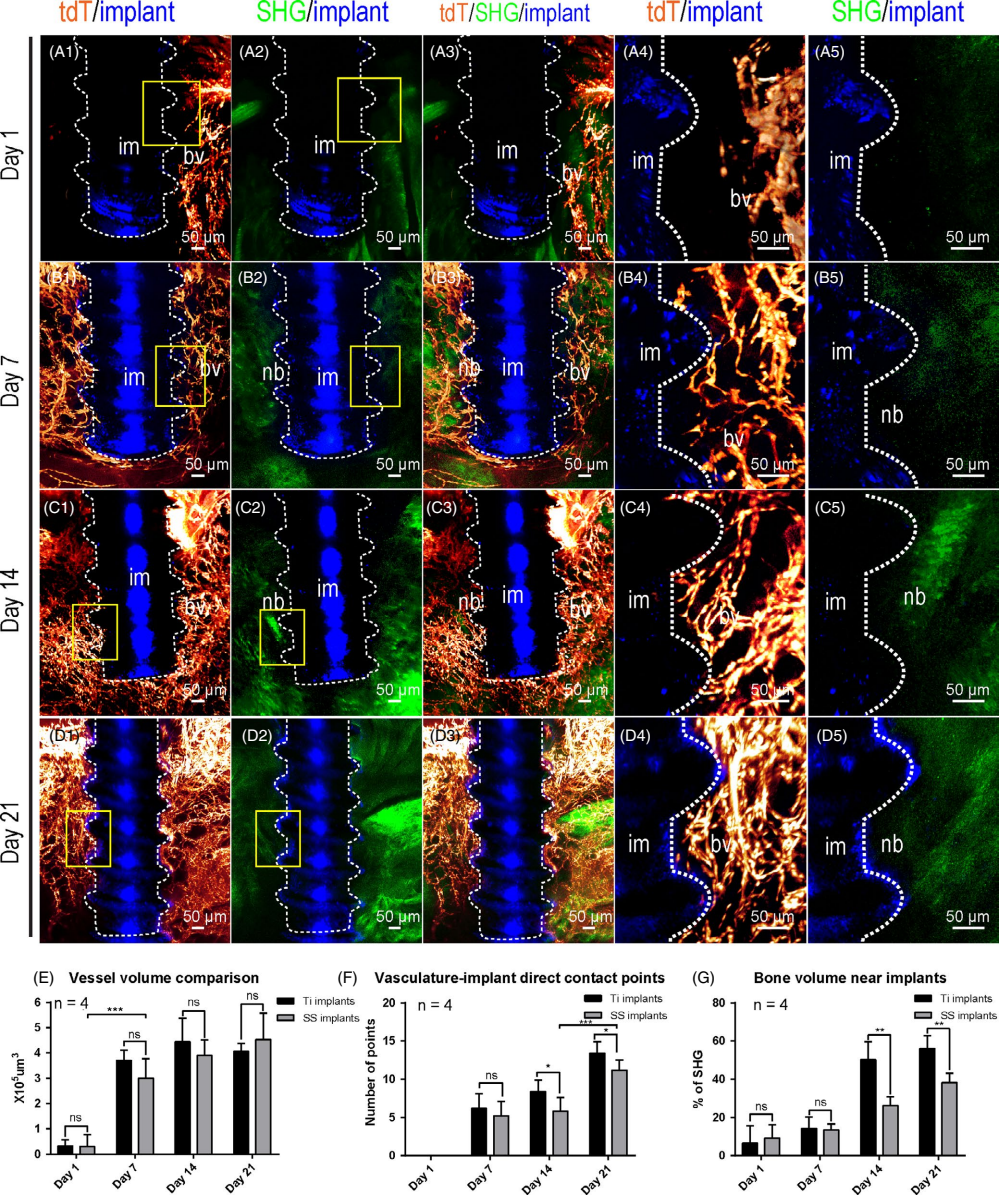
Three‐dimensional quantitative analysis shows comparable angiogenesis but reduced osteogenesis on the surface of stainless steel implants than on titanium implants surface. Adult *Cdh5‐Cre^ERT2^*; *Ai4* mice (2 mo of age) were used for stainless steel implant placement. Samples with stainless steel implants were cleared with decalcified polyethylene glycol (PEG)‐associated solvent system method and imaged in the same way as for the titanium implants. Optical stacks of 150 μm thickness were acquired to demonstrate blood vessels (tdTomato, red) surrounding the interface between implant (reflection image, blue) and mandible bone (second harmonic generation, green). (A1‐A3) Images were acquired with 10× objective 1 d after implant placement. A4‐A5, Boxed areas in A1 and A2 were re‐imaged with a 25× objective. B1‐B3, Images were acquired with 10× objective 1 wk after implant placement. B4‐B5, Boxed areas in B1 and B2 were re‐imaged with a 25× objective. C1‐C3, Images were acquired with 10× objective 2 wk after implant placement. C4‐C5, Boxed areas in C1 and C2 were re‐imaged with a 25× objective. D1‐D3, Images were acquired with 10× objective 3 wk after implant placement. D4‐D5, Boxed areas in D1 and D2 were re‐imaged with a 25× objective. E, Comparison of total blood vessel volumes around Ti and SS implants at different time points. The quantification results for Ti implants were from Figure 2. n = 4. F, Comparison of percentiles of bone matrix volume near Ti and SS implants at different time points. n = 4. G, Comparison of number of direct contact points between the implant and blood vessels at different time points. n = 4. Scale bars, 100 μm. bv, blood vessels; im, implant; nb, new bone

Next, we evaluated the angiogenesis and osteogenesis processes at the stainless steel implant‐tissue interface (Figure 4). Similar to the titanium implant, 1 day after implant placement, few blood vessels or bones were visualized near the steel implant surface (Figure 5A1‐A5). One week after implant placement, enriched blood vessels, but little bone, were visualized near the implant surface (Figure 5B1‐B5). Two weeks after implant placement, enriched blood vessels were detected at the interface. The amount of bone close to the implant surface remained low. Three weeks later, the density of blood vessels continued to increase. Stronger SHG signal could be detected, but still little was found to contact directly with implant surface. Quantitative comparison was made between the titanium and stainless steel implants using “vessel volume, bone volume and vasculature‐implant contact points” as the index (Figure 5E‐G). No significant difference was found among blood vessel volumes at different time points between these two types of implant. The slightly less vasculature‐implant direct contact points in stainless steel implants group suggest stainless steel might be less amenable than titanium. The bone amount surrounding the stainless steel implant was much lower than that of titanium implants at Day 14 and Day 21. µCT analysis also showed similar bone density change surrounding stainless steel implants (Figure S3E‐I).

### 3.7 3‐D imaging of non‐decalcified samples revealed distinct calcium precipitation activities surrounding titanium or stainless steel implants

**Figure 6:**
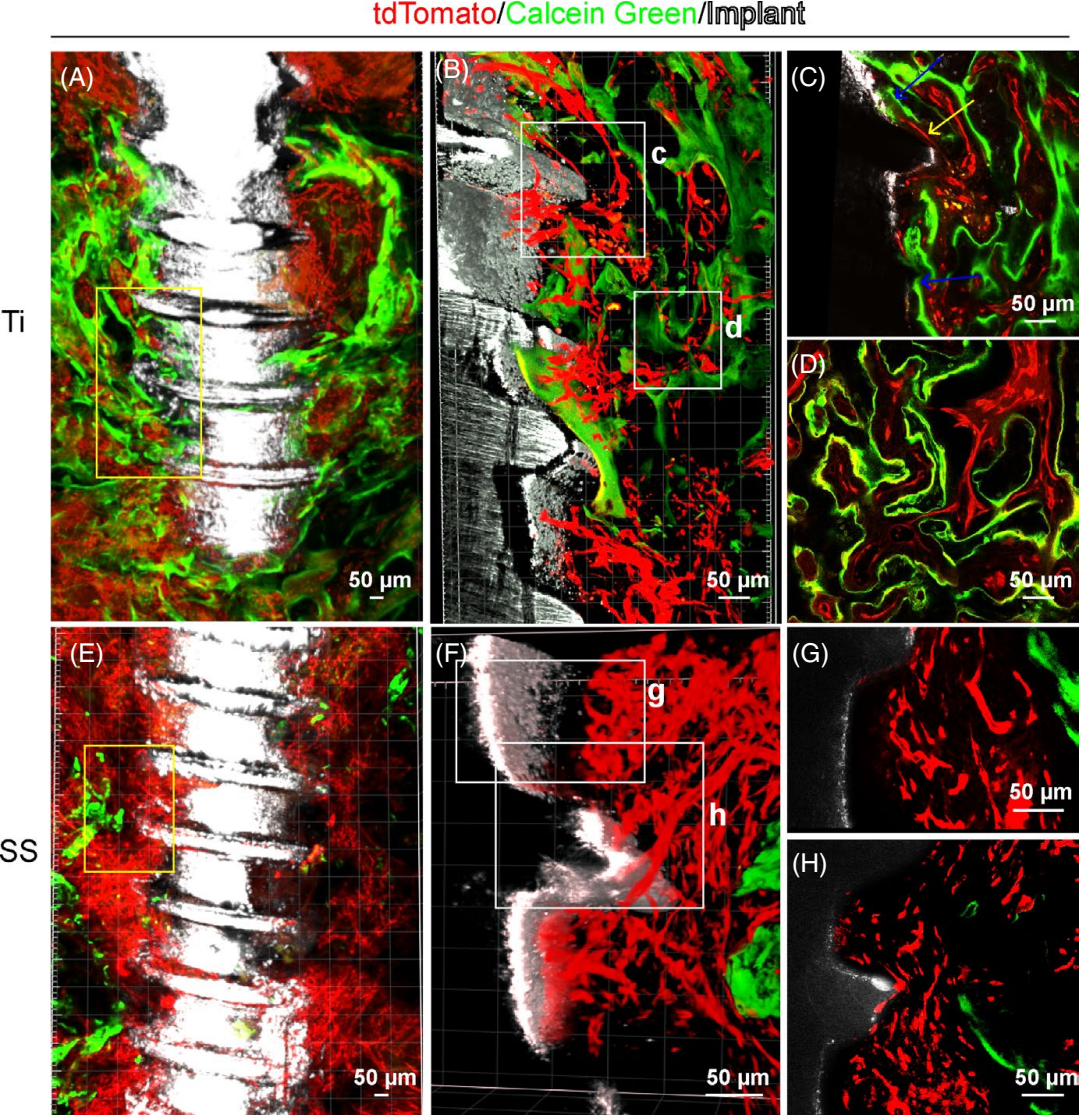
Calcein green labelling indicates distinct calcium precipitation activity on surfaces of stainless steel or titanium implants. Adult *Cdh5‐Cre^ERT2^*; *Ai4* mice (6 wk of age) were used for implant placement and calcein green labelling. Mandible samples were processed following polyethylene glycol (PEG)‐associated solvent system method without decalcification treatment. Images were acquired with 10× (A and E) or 25× (B, C, D, F, G and H) objective on a ZEISS two‐photon microscope. A, An optical stack of 150 μm showing enriched blood vessels (red) and calcium precipitation activity (green) near titanium implant (white). B, Boxed area in (A) was re‐imaged with a 25× objective. C, Optical section of boxed area in (B). Yellow arrows show blood vessels in direct contact with the implant surface. Blue arrows indicate calcium deposition at the implant surface. D, Optical section of boxed area in (B) showing association of angiogenesis and osteogenesis near the implant surface. E, An optical stack of 150 μm showing blood vessels and calcium precipitation activity near the stainless steel implant. F, Boxed area in (E) was re‐imaged with a 25× objective. G, Optical section of boxed area in (F) shows abundant blood vessels near the stainless steel implant. H, Optical section of boxed area in (F) shows sparse calcium precipitation near the stainless steel implant surface

To further test the mineral deposition activity near these two implant surfaces, we performed calcein green labelling to mark the calcium precipitation regions. Three weeks after titanium implant placement, abundant blood vessels were detected surrounding the titanium implant surface (Figure 6A). Strong calcein green signal was detected suggesting highly active osteogenic process (Figure 6A). Enlarged images indicated existence of calcein green signal immediately on the titanium implant surface in close association with blood vessels (Figure 6B‐D). In contrast, little calcein green signal was detected surrounding the stainless steel implants 3 weeks after surgery (Figure 6E). Although plenty of blood vessels were visualized on the stainless steel implant surface (Figure 6F‐H), little calcein green signal was detected surrounding blood vessels near the implant surface (Figure 6G,H). Active calcein green signal was only detected at a distance from the stainless steel implant surface (Figure 6E).

## 4 DISCUSSION

Tissue clearing technique has been widely used in neuroscience research for years. Our current study is the first of its applications on investigating the implant‐tissue interface. Capability of the PEGASOS method to clear both hard and soft tissue organs plays critical roles for such application. Bones and teeth could be cleared to nearly invisible after treatment. In addition, the treatment procedure preserves endogenous fluorescence better than other solvent‐based clearing methods, including 3DISCO, uDISCO and FluoClear.^30^ The fluorescence preservation is critical for acquiring multi‐channel 3‐D images after tissue clearing and enables applications of multiple transgenic mouse models to investigate implant‐tissue interface. Combining with fluorescent reporter mouse lines, multi‐colour 3‐D investigation of implant‐bone interface is becoming possible. Inside structures and implant surface can be directly visualized with a regular confocal or two‐photon microscope without sectioning.

The depth of imaging is mainly determined by tissue transparency and working distance of microscope objectives. Complete transparency of hard tissue is challenging due to the presence of hydroxyapatite crystal, colourized bone marrow and, most importantly, different refractive index (RI) among different tissue components. The favourable transparency achieved with PEGASOS can be attributed to the combination of decalcification, decolourization, delipidation and the high RI (1.543) of BB‐PEG clearing medium, which is close to the average RI (1.53) of decalcified bone.^30^, ^42^, ^43^ In our current work, with PEGASOS decalcification clearing method, we were able to acquire 3‐D images up to 800 µm depth. Image resolution dropped when the depth is over 1 mm in the bone (data not shown). In our other study, we were able to acquire images up to 3 mm depth on a cleared dog tibia bone sample.^30^

Partial transparency was achieved for non‐decalcified mandible bone sample, which can be attributed to high RI of the BB‐PEG clearing medium. Images with satisfactory resolution could be achieved up to 300 µm depth, which is sufficient for our research purposes. Such capability further broadens the application of PEGASOS in hard tissue research because decalcification treatment compromises calcein green or doxycycline incorporation signal. Although 3‐D dynamic labelling was tested using BABB clearing method,^43^ PEGASOS remains the only technique capable of combining dynamic labelling with endogenous fluorescent labels due to its preservation of GFP or tdTomato signal.

It remains a concern whether decalcification treatment compromises the implant‐bone interface. Our study indicated that decalcification treatment has no detectable damage to the implant‐bone interface. PEGASOS clearing treatment also has no detectable effect on the interface. Decalcification treatment significantly improves bone and dental tissue transparency and has no impact on endogenous fluorescence. Therefore, we highly recommend incorporation of decalcification treatment in future tissue clearing studies on bone and dental tissues.

Second harmonic generation arises from type I collagen independently of GFP labelling.^44^, ^45^ A multi‐photon microscope is required for its detection.^46^, ^47^ Application of SHG on visualizing bone provides multiple advantages over conventional X‐RAY technique. First of all, SHG signal can be detected on both non‐decalcified and decalcified samples. Second, SHG signal can be combined with other fluorescent labels. Third, resolution of an SHG signal image can be easily improved by using a high‐magnification objective. If needed, an SHG image can be acquired with a 40×/1.3NA objective to achieve ~0.2 µm lateral resolution, which is far better than any current µCT equipment. In addition, SHG image has no halation surrounding metal implant, which is a common artefact for µCT analysis.^48^, ^49^

Although angiogenesis is known to be a prerequisite for osteogenesis, our study indicates that successful angiogenesis does not guarantee successful osteogenesis. Titanium and stainless steel implants possess different capability on supporting osteogenesis and implant osseointegration. It is possible that stem cell populations supporting bone healing and osseointegration were differentially affected near the surfaces of titanium or stainless steel implant. Further investigation is needed to test this hypothesis.

Polyethylene glycol‐associated solvent system method has its own limitations when imaging implant‐tissue interface. First of all, PEGASOS treatment leads to differential shrinkage among soft and hard tissues, which may cause anisotropic distortion for organs composed of multiple tissue types. Second, although PEGASOS protects endogenous fluorescence better than other solvent‐based clearing methods, it still compromises GFP or tdTomato fluorescence intensity significantly. Third, auto‐fluorescence from bone marrow and muscle tissue may increase after clearing treatment, which deteriorates signal/noise ratio especially in deep region.^30^ Our laboratory is working to improve PEGASOS method to overcome these limitations.

In the current study, we introduced the application of the PEGASOS tissue clearing method on studying bone‐implant interface. By using *Cdh5‐Cre^ERT2^*; *Ai14* mouse model to label vascular endothelium, we demonstrated the angiogenesis and osteogenesis processes at the implant‐bone interface. We showed that both titanium and stainless steel implants support angiogenesis, but only titanium implants support osteogenesis and osseointegration. 3‐D multi‐channel images of calcein green labelling with other signals further confirmed the distinct osteogenic activities on surfaces of two different types of implant. PEGASOS tissue clearing–based deep imaging provides a valuable new tool for studying tissue‐material interactions and will help researchers to design better strategies for tissue engineering and regeneration.

## CONFLICT OF INTEREST

The authors declare no conflict of interest.

## AUTHOR CONTRIBUTION

HZ designed and supervised the study. YY performed most of the experiments. YM assisted with the animal surgery and statistical analysis. DJ, SZ and WL assisted with the image acquisition. JF and J.L provided critical comments. The manuscript was written by HZ, YY and JW. All authors gave final approval and agreed to be accounted for all aspects of the work.

## References

[1] Raphel J , Holodniy M , Goodman SB , Heilshorn SC . Multifunctional coatings to simultaneously promote osseointegration and prevent infection of orthopaedic implants. Biomaterials. 2016;84:301‐314. 26851394 10.1016/j.biomaterials.2016.01.016 PMC4883578

[2] Bai L , Liu Y , Du Z , et al. Differential effect of hydroxyapatite nano‐particle versus nano‐rod decorated titanium micro‐surface on osseointegration. Acta Biomater. 2018;76:344‐358. 29908975 10.1016/j.actbio.2018.06.023

[3] Chen W , Xu K , Tao B , et al. Multilayered coating of titanium implants promotes coupled osteogenesis and angiogenesis in vitro and in vivo. Acta Biomater. 2018;74:489‐504. 29702291 10.1016/j.actbio.2018.04.043

[4] Breding K , Jimbo R , Hayashi M , Xue Y , Mustafa K , Andersson M . The effect of hydroxyapatite nanocrystals on osseointegration of titanium implants: an in vivo rabbit study. Int J Dent. 2014;2014:171305. 24563651 10.1155/2014/171305 PMC3915854

[5] Al Subaie AE , Eimar H , Abdallah MN , et al. Anti‐VEGFs hinder bone healing and implant osseointegration in rat tibiae. J Clin Periodontol. 2015;42(7):688‐696. 26073407 10.1111/jcpe.12424

[6] Yu Y , Jin G , Xue Y , Wang D , Liu X , Sun J . Multifunctions of dual Zn/Mg ion co‐implanted titanium on osteogenesis, angiogenesis and bacteria inhibition for dental implants. Acta Biomater. 2017;49:590‐603. 27915020 10.1016/j.actbio.2016.11.067

[7] Hu XF , Wang L , Xiang G , Lei W , Feng YF . Angiogenesis impairment by the NADPH oxidase‐triggered oxidative stress at the bone‐implant interface: critical mechanisms and therapeutic targets for implant failure under hyperglycemic conditions in diabetes. Acta Biomater. 2018;73:470‐487. 29649637 10.1016/j.actbio.2018.04.008

[8] Saran U , Gemini Piperni S , Chatterjee S . Role of angiogenesis in bone repair. Arch Biochem Biophys. 2014;561:109‐117. 25034215 10.1016/j.abb.2014.07.006

[9] Scarano A , Perrotti V , Artese L , et al. Blood vessels are concentrated within the implant surface concavities: a histologic study in rabbit tibia. Odontology. 2014;102(2):259‐266. 23783569 10.1007/s10266-013-0116-3

[10] Schneider P , Krucker T , Meyer E , et al. Simultaneous 3D visualization and quantification of murine bone and bone vasculature using micro‐computed tomography and vascular replica. Microsc Res Tech. 2009;72(9):690‐701. 19360841 10.1002/jemt.20720

[11] Núñez JA , Goring A , Hesse E , et al. Simultaneous visualisation of calcified bone microstructure and intracortical vasculature using synchrotron X‐ray phase contrast‐enhanced tomography. Sci Rep. 2017;71(1):13289. 10.1038/s41598-017-13632-5 PMC5643345 29038597

[12] Huang C , Ness VP , Yang X , et al. Spatiotemporal analyses of osteogenesis and angiogenesis via intravital imaging in cranial bone defect repair. J Bone Miner Res. 2015;30(7):1217‐1230. 25640220 10.1002/jbmr.2460 PMC4618698

[13] He T , Cao C , Xu Z , et al. A comparison of micro‐CT and histomorphometry for evaluation of osseointegration of PEO‐coated titanium implants in a rat model. Sci Rep. 2017;7(1):16270. 29176604 10.1038/s41598-017-16465-4 PMC5701240

[14] Mouraret S , Hunter DJ , Bardet C , Brunski JB , Bouchard P , Helms JA . A pre‐clinical murine model of oral implant osseointegration. Bone. 2014;58:177‐184. 23886841 10.1016/j.bone.2013.07.021 PMC4962868

[15] von Wilmowsky C , Moest T , Nkenke E , Stelzle F , Schlegel KA . Implants in bone: part II. Research on implant osseointegration: material testing, mechanical testing, imaging and histoanalytical methods. Oral Maxillofac Surg. 2014;18(4):355‐372. 23430020 10.1007/s10006-013-0397-2

[16] Babu RA , Ogle O . Tissue response: biomaterials, dental implants, and compromised osseous tissue. Dent Clin North Am. 2015;59(2):305‐315. 25835795 10.1016/j.cden.2014.10.010

[17] Xiong H , Zhou Z , Zhu M , et al. Chemical reactivation of quenched fluorescent protein molecules enables resin‐embedded fluorescence microimaging. Nat Commun. 2014;5:3992. 24886825 10.1038/ncomms4992 PMC4059927

[18] Daroff RB , Aminoff MJ . Encyclopedia of the Neurological Sciences. Oxford, UK: Elsevier Science; 2014.

[19] Neu CP , Novak T , Gilliland KF , Marshall P , Calve S . Optical clearing in collagen‐ and proteoglycan‐rich osteochondral tissues. Osteoarthritis Cartilage. 2015;23(3):405‐413. 25454370 10.1016/j.joca.2014.11.021 PMC4339456

[20] Alt V , Kogelmaier DV , Lips KS , et al. Assessment of angiogenesis in osseointegration of a silica‐collagen biomaterial using 3D‐nano‐CT. Acta Biomater. 2011;7(10):3773‐3779. 21723963 10.1016/j.actbio.2011.06.024

[21] Treweek JB , Chan KY , Flytzanis NC , et al. Whole‐body tissue stabilization and selective extractions via tissue‐hydrogel hybrids for high‐resolution intact circuit mapping and phenotyping. Nat Protoc. 2015;10(11):1860‐1896. 26492141 10.1038/nprot.2015.122 PMC4917295

[22] Schwarz MK , Scherbarth A , Sprengel R , Engelhardt J , Theer P , Giese G . Fluorescent‐protein stabilization and high‐resolution imaging of cleared, intact mouse brains. PLoS ONE. 2015;10(5):e0124650. 25993380 10.1371/journal.pone.0124650 PMC4439039

[23] Pan C , Cai R , Quacquarelli FP , et al. Shrinkage‐mediated imaging of entire organs and organisms using uDISCO. Nat Methods. 2016;13(10):859‐867. 27548807 10.1038/nmeth.3964

[24] Chung K , Deisseroth K . CLARITY for mapping the nervous system. Nat Methods. 2013;10(6):508‐513. 23722210 10.1038/nmeth.2481

[25] Kubota SI , Takahashi K , Nishida J , et al. Whole‐body profiling of cancer metastasis with single‐cell resolution. Cell Rep. 2017;20(1):236‐250. 28683317 10.1016/j.celrep.2017.06.010

[26] Oren R , Fellus‐Alyagor L , Addadi Y , et al. Blood and lymphatic vessels imaging (WOBLI). Sci Rep. 2018;8(1):1412. 29362484 10.1038/s41598-018-19663-w PMC5780490

[27] Tainaka K , Kuno A , Kubota SI , Murakami T , Ueda HR . Chemical principles in tissue clearing and staining protocols for whole‐body cell profiling. Annu Rev Cell Dev Biol. 2016;32:713‐741. 27298088 10.1146/annurev-cellbio-111315-125001

[28] Cai R , Pan C , Ghasemigharagoz A , et al. Panoptic vDISCO imaging reveals neuronal connectivity, remote trauma effects and meningeal vessels in intact transparent mice. bioRxiv. 2018;374785.

[29] Renier N , Wu Z , Simon David J , Yang J , Ariel P , Tessier‐Lavigne M . iDISCO: a simple, rapid method to immunolabel large tissue samples for volume imaging. Cell. 2014;159(4):896‐910. 25417164 10.1016/j.cell.2014.10.010

[30] Jing D , Zhang S , Luo W , et al. Tissue clearing of both hard and soft tissue organs with the PEGASOS method. Cell Res. 2018;28(8):803‐818. 29844583 10.1038/s41422-018-0049-z PMC6082844

[31] Hama H , Kurokawa H , Kawano H , et al. Scale: a chemical approach for fluorescence imaging and reconstruction of transparent mouse brain. Nat Neurosci. 2011;14(11):1481‐1488. 21878933 10.1038/nn.2928

[32] Kuwajima T , Sitko AA , Bhansali P , Jurgens C , Guido W , Mason C . ClearT: a detergent‐ and solvent‐free clearing method for neuronal and non‐neuronal tissue. Development. 2013;140(6):1364‐1368. 23444362 10.1242/dev.091844 PMC3912244

[33] Ke MT , Nakai Y , Fujimoto S , et al. Super‐resolution mapping of neuronal circuitry with an index‐optimized clearing agent. Cell Rep. 2016;14(11):2718‐2732. 26972009 10.1016/j.celrep.2016.02.057

[34] Tainaka K , Kubota SI , Suyama TQ , et al. Whole‐body imaging with single‐cell resolution by tissue decolorization. Cell. 2014;159(4):911‐924. 25417165 10.1016/j.cell.2014.10.034

[35] Murakami TC , Mano T , Saikawa S , et al. A three‐dimensional single‐cell‐resolution whole‐brain atlas using CUBIC‐X expansion microscopy and tissue clearing. Nat Neurosci. 2018;21(4):625‐637. 29507408 10.1038/s41593-018-0109-1

[36] Woo J , Lee M , Seo JM , Park HS , Cho YE . Optimization of the optical transparency of rodent tissues by modified PACT‐based passive clearing. Exp Mol Med. 2016;48(12):e274. 27909337 10.1038/emm.2016.105 PMC5192069

[37] Greenbaum A , Chan KY , Dobreva T , et al. Bone CLARITY: clearing, imaging, and computational analysis of osteoprogenitors within intact bone marrow. Sci Transl Med. 2017;9(387):eaah6518. 28446689 10.1126/scitranslmed.aah6518

[38] Eilken HM , Dieguez‐Hurtado R , Schmidt I , et al. Pericytes regulate VEGF‐induced endothelial sprouting through VEGFR1. Nat Commun. 2017;8(1):1574. 29146905 10.1038/s41467-017-01738-3 PMC5691060

[39] Boas FE , Fleischmann D . CT artifacts: causes and reduction techniques. Imaging Med. 2012;4(2):229‐240.

[40] Vandeweghe S , Coelho PG , Vanhove C , Wennerberg A , Jimbo R . Utilizing micro‐computed tomography to evaluate bone structure surrounding dental implants: a comparison with histomorphometry. J Biomed Mater Res B Appl Biomater. 2013;101(7):1259‐1266. 23661363 10.1002/jbm.b.32938

[41] van Gaalen SM , Kruyt MC , Geuze RE , de Bruijn JD , Alblas J , Dhert WJ . Use of fluorochrome labels in in vivo bone tissue engineering research. Tissue Eng Part B Rev. 2010;16(2):209‐217. 19857045 10.1089/ten.TEB.2009.0503

[42] Ascenzi A , Fabry C . Technique for dissection and measurement of refractive index of osteones. J Biophys Biochem Cytol. 1959;6(1):139‐142. 13673068 10.1083/jcb.6.1.139 PMC2229768

[43] Berke IM , Miola JP , David MA , Smith MK , Price C . Seeing through musculoskeletal tissues: improving in situ imaging of bone and the lacunar canalicular system through optical clearing. PLoS ONE. 2016;11(3):e0150268. 26930293 10.1371/journal.pone.0150268 PMC4773178

[44] Ambekar R , Chittenden M , Jasiuk I , Toussaint Jr KC . Quantitative second‐harmonic generation microscopy for imaging porcine cortical bone: comparison to SEM and its potential to investigate age‐related changes. Bone. 2012;50(3):643‐650. 22155019 10.1016/j.bone.2011.11.013

[45] Chen X , Nadiarynkh O , Plotnikov S , Campagnola PJ . Second harmonic generation microscopy for quantitative analysis of collagen fibrillar structure. Nat Protoc. 2012;7(4):654‐669. 22402635 10.1038/nprot.2012.009 PMC4337962

[46] Fonseca JE . Bone biology: from macrostructure to gene expression. Medicographia. 2012;34:142‐148.

[47] Tokarz D , Cisek R , Wein MN , et al. Intravital imaging of osteocytes in mouse calvaria using third harmonic generation microscopy. PLoS ONE. 2017;12(10):e0186846. 29065178 10.1371/journal.pone.0186846 PMC5655444

[48] Butz F , Ogawa T , Chang TL , Nishimura I . Three‐dimensional bone‐implant integration profiling using micro‐computed tomography. Int J Oral Maxillofac Implants. 2006;21(5):687‐695. 17066629

[49] Swain MV , Xue J . State of the art of Micro‐CT applications in dental research. Int J Oral Sci. 2009;1(4):177‐188. 20690421 10.4248/IJOS09031 PMC3470105

