# Supplementary material for: 3‐dimensional visualization of implant‐tissue interface with the polyethylene glycol associated solvent system tissue clearing method
Source: Cell Prolif. 2019 Feb 3;52(3):e12578. doi: 10.1111/cpr.12578 (PMC6536405; doi:10.1111/cpr.12578)
Supplement: Supplementary file 1 [file CPR-52-e12578-s001.docx]

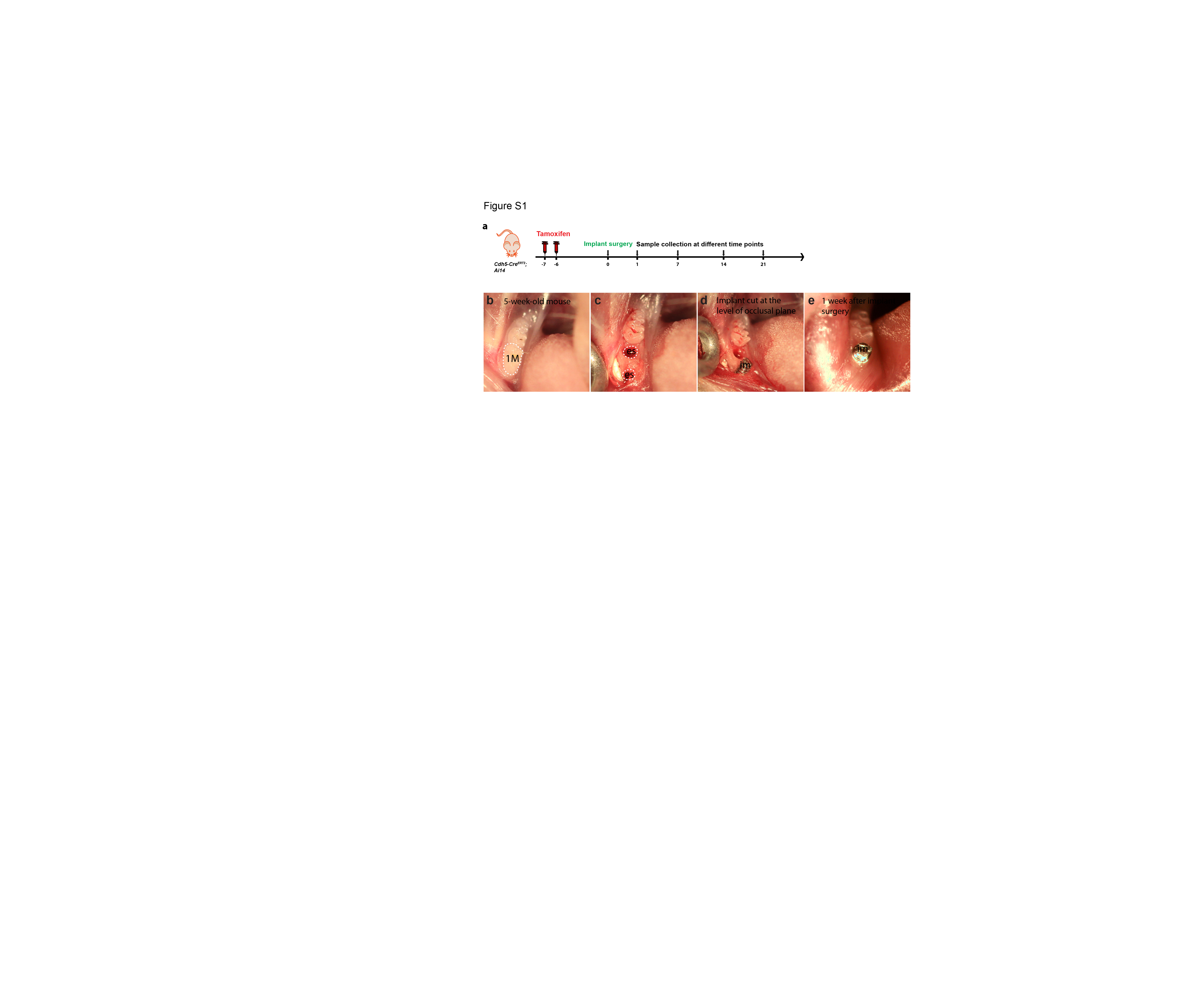


**Figure S1. Description of experimental scheme and animal surgery.**

(A) Experiment scheme: time points for tamoxifen injection, implant surgery and sample harvest.

(B-E) Procedure of implant placement surgery: (B, C) Mouse mandibular first molar was extracted. (D) The implant was screwed into the extraction socket manually and cut off at the level of the occlusal plane. (E) Soft tissue around the implant was healed 7 days after surgery. 1M: First molar; es: extraction socket; im: implant.


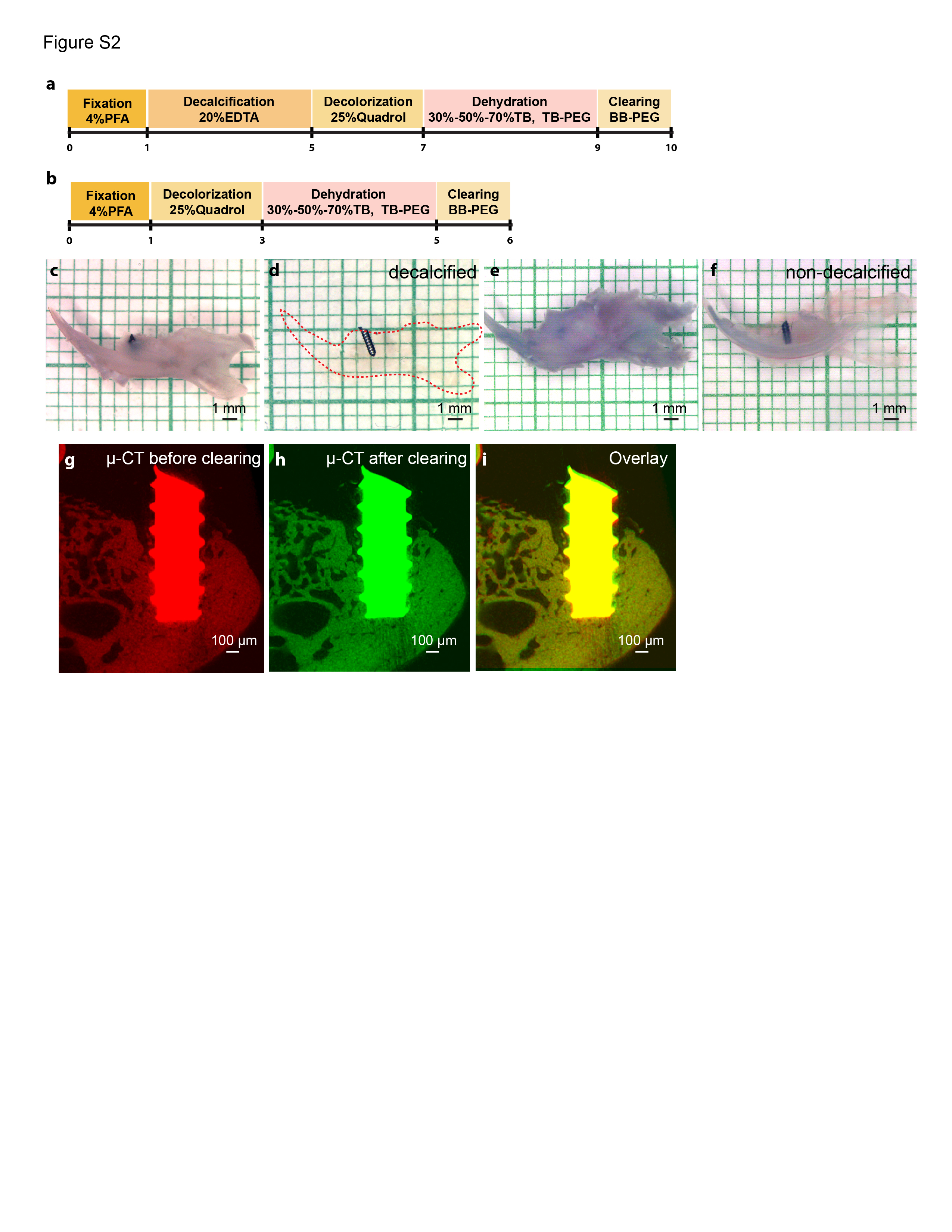


**Figure S2. PEGASOS with decalcification turns all tissues surrounding the implant transparent. Non-decalcified PEGASOS cleared hard tissue with partial transparency and preserves intact bone structure.**

(A, B) Brief description of the PEGASOS procedure with (A) or without (B) decalcification treatment for clearing a mouse mandible.

(C-F) Images of mandible samples before (C, E) and after (D, F) PEGASOS clearing treatment. Scale bars, 1*mm*.

(G-I) Comparison of *μ*-CT images of a mandible sample before (G) and after (H) clearing with non-decalcified PEGASOS method. The two images were overlaid in (I). Scale bars, 100 *μm.*


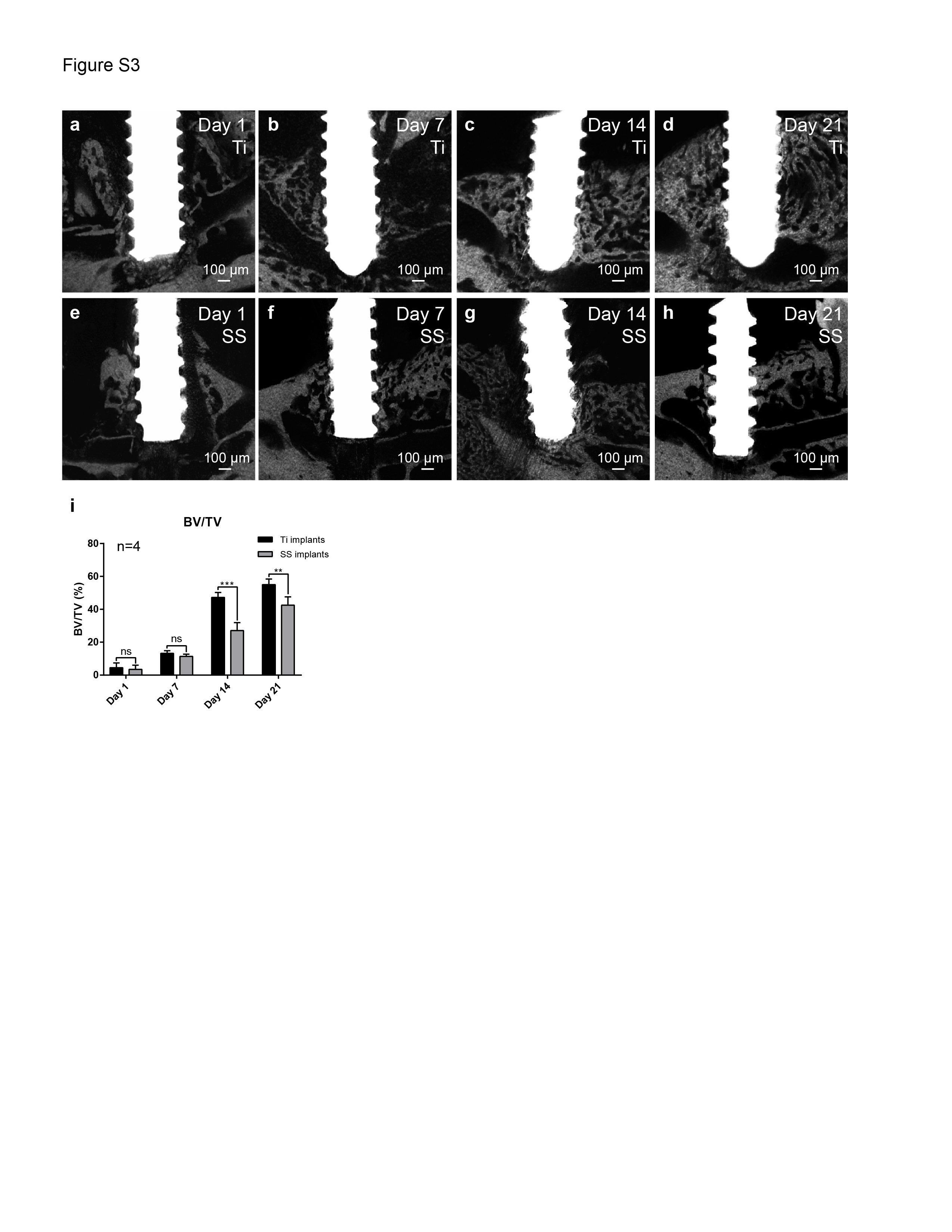


**Figure S3. Micro-CT analysis of peri-implant bone.**

(A-D) Micro-CT images of titanium implants and supporting tissues on post surgery day 1, 7, 14 and 21.

(E-H) Micro-CT images of stainless steel implants and supporting tissues on post surgery day 1, 7 and 14 and 21.

(I) Quantified comparison of BV/TV near titanium and stainless steel implants.

Scale bars, 100*μm.*
